# Plasma concentrations of cortisol and PGF_2α _metabolite in Danish sows during mating, and intrauterine and conventional insemination

**DOI:** 10.1186/1751-0147-49-36

**Published:** 2007-12-05

**Authors:** Mattias Norrby, Mads T Madsen, Charlotte Borg Alexandersen, Hans Kindahl, Andrzej Madej

**Affiliations:** 1 Department of Anatomy, Physiology and Biochemistry, Swedish University of Agricultural Sciences, PO Box 7011, SE-750 07 Uppsala, Sweden; 2 Danish Pig Production, Axeltorv 3, DK-1609 Copenhagen, Denmark; 3 65 Rigtrupvej, 8370 Hadsten, Denmark; 4 Department of Clinical Sciences, Division of Reproduction, Swedish University of Agricultural Sciences, PO Box 7054, SE-750 07 Uppsala, Sweden

## Abstract

**Background:**

The aims of the present work was to study whether there are any relationships between cortisol and PG-metabolite in mated sows or inseminated with the intrauterine technique and compare these to changes occurring in conventionally inseminated sow.

**Methods:**

Thirty three crossbred sows (Danish Landrace × Danish Large White) were fitted with jugular vein catheters through vena auricularis from one of the ears. The sows were randomly divided into three groups (Boar-, IUI- and AI-group) and blood samples were collected before, during and after service. In a final evaluation only 25 sows that became pregnant and farrowed piglets at full term were used.

**Results:**

Cortisol concentrations increased in all groups but Boar-group (n = 8) had a significantly higher cortisol during 10 to 20 min after service than sows in AI-group (n = 8). In mated sows cortisol concentrations peaked at 15 minutes after service. The Boar-group (n = 8) showed no ascending PG-metabolite levels during the whole experiment, while both IUI- and AI-groups (n = 9 and n = 8, respectively) had a 2.5-fold increase in PG-metabolite 15 minutes after service.

**Conclusion:**

In conclusion, mating of sows by a boar results in a greater increase of cortisol than AI and without an elevation of PG-metabolite levels, which was seen in both the inseminated groups. It was also demonstrated that IUI-group had an earlier significant increase of PG-metabolite levels than sows inseminated conventionally. Further investigation using different semen extenders or even different type of insemination catheters might be helpful in understanding the reason for an immediate increase of PG-metabolite after insemination but not after mating.

## Background

At mating, as well as during conventional artificial insemination (AI), semen is deposited into the cervical canal of the female. Semen transport along the uterine horns is elicited by myometrial contractions and a certain number of spermatozoa reach the uterotubal junction (UTJ) and the first part of isthmus. In isthmus a sperm reservoir forms in order to (i) escape reflux and phagocytosis in the uterine lumen and (ii) ensure that sufficient numbers of potentially fertile spermatozoa are present at the site of fertilization when oocytes are released [1]. Prostaglandin (PG) F_2α _is known to be involved in myometrial contractions [2-4]. Langendijk et al. [5] demonstrated that both suppression and stimulation of uterine contractions adversely affect fertilization in sows. Measurement of cortisol in blood is a traditional methodology for monitoring and evaluating stress in pigs, even though it is known that cortisol is not solely a stress-mediated hormone. Glucocorticoids are complex anti-inflammatory drugs and are thought to alter gene expression by as much as 1% in their target cells [6]. An increase of cortisol in response to mating appears to be a normal part of the mating process in the female pig [7]. Our previous studies indicated an interaction between cortisol and prostaglandin F_2α _metabolite (PG-metabolite) in sows during AI depending on the type of housing [8]. Madsen et al. [9] reported that AI resulted in an increase of PG-metabolite in sows and that no increases were seen in sows mated by boar. Recently, the elevation of PG-metabolite has been seen also in sows inseminated IUI [10]. The aims of the present work were to study whether there are any relationships between cortisol and PG-metabolite in mated sows or inseminated with the intrauterine technique and compare these to changes occurring in conventionally inseminated sows.

## Methods

### Animals

The experiments were performed, during July and August, in a herd (Grønhøj, Denmark) with approximately 600 sows managed with one-week batch operations. Twenty to 25 sows were moved weekly after weaning, on Wednesdays, from the farrowing house to the mating house. Six to eight sows were catheterised every Friday but some of them were not included due to problems with the catheter. The remaining sows were randomly divided into the three experimental groups. All sows were held in crates. Thirty-three crossbreed sows with parity ranging from 1 to 7 were used in the experiment. The parity of the selected sows was 3.7 ± 1.7 (mean ± SD) with no differences between groups. Three groups with 11 sows in each group were formed. Sows were served by boar (Boar-group), inseminated intrauterinally (IUI-group) or inseminated conventionally (AI-group). The care of animals and the design of the experiment were approved by The Animal Experimentation Inspectorate, Ministry of Justice, Denmark" (J.nr. 1999/561-234) and by the Danish Medicines Agency, Denmark (J.nr. 2512-8435)

### Mating and insemination

During the weekend, the staff checked the sows for standing oestrus by means of the back pressure test and the riding test. The sows had the opportunity for nose contact with a boar. The sows were served either on Mondays or Tuesdays during their standing oestrus. Two Danish Duroc boars were used, evenly distributed, to serve the sows in the Boar-group. All sows were served during the first oestrus after weaning. The sows in the Boar-group were moved to the boar for mating while AI- and IUI-group were served in the crate. Regardless of insemination method, the sow was stimulated after a 5-point stimulation plan. The 5-point stimulation plan included the back pressure test after manipulation of the abdominal area, the area under vulva, the inguinal area and the pelvic area. For intrauterine insemination an insemination catheter "Deep goldenpig" (IMV Technologies, L'Aigle, France) was used. The catheter was inserted as a conventional insemination catheter by placing it counter clockwise into the distal cervix. Then a soft inner tube, 4 mm in diameter, was gently pushed through the cervix with a minimal pressure, with the inner tube extending 20 cm beyond the tip of the outer tube. When stopped by resistance the AI-technician waited for the cervix to relax so insertion could continue. Both IUI- and AI-groups were inseminated with 80 ml extended semen in EDTA, both purchased from Hatting K/S, Denmark. The semen was a mixture from 2–10 boars of the Danish Duroc breed. Each dose of semen contained 2 × 10^9 ^progressive motile spermatozoa.

### Venous cannulation

The sows were catheterized in vena jugularis externa through vena auricularis on Fridays (two days after weaning). An intravenous catheter with one short outer cannula and one long soft inner catheter (B|Braun, Cavafix^® ^Certo^®^, catheter: 1.1 × 1.7 mm/16 G, length 45 cm. Cannula: 1.8 × 2.35 mm/14 G, length 7 cm, flow rate 36 ml/min) was used. Fifteen to 20 minutes before catheterization sows were given 2 mg/kg body weight of Stresnil^® ^(Azaperone 40 mg/ml, Bayer) intra-muscularly. The sows were fixed with a nose sling during catheterization. The ear was shaved on both sides so the catheter could be fixed with plaster. Then the cannula and the catheter were inserted and immediately afterwards the catheter was filled with heparinised saline (1 ml heparin/100 ml isotonic saline) to avoid clotting in the catheter. After the service, the catheter was removed and an inspection of the ear carried out for any sign of inflammation.

### Blood sampling

On the day of the service, which includes both stimulation and insemination or mating, 10 ml blood samples were taken 10 and 5 minutes before the service, when the service started, and 1, 2, 3, 4, 6, 8, 10, 15, 20, 25, 30, 35, 40, 45, 50 minutes after the service started. The first 4 minutes (minutes 0 to 4) is stimulation either by the boar or human stimulation. Due to the blood flow in the cannula, blood samples were taken e.g. between 1 and 2 minutes, 2 to 3 minutes etc. The blood was collected in heparinized tubes containing 0.5 ml Trasylol^® ^(Aprotinin 10000 KIE/ml, Bayer). The tubes were immediately chilled on ice and then centrifuged at 4°C at 3200 rpm (1000 × g) for 10 minutes. After centrifugation, plasma from each sample was divided in two 5 ml polypropylene capped containers. The polypropylene containers were immediately placed in a -20°C freezer. Afterwards, plasma samples were stored at -70°C until analyzed. Between samples with 5 minutes interval, the catheters were flushed with heparinized saline solution to avoid coagulation. Before the next sample was collected, the heparinized saline solution in the catheter was discarded.

### Cortisol analyses

Plasma cortisol was measured by radioimmunoassay (Coat-A-Count Cortisol: Diagnostic Products Corporation, Los Angeles, CA, USA) used according to the manufacturer's recommendations and validation for porcine plasma [11]. The intra-assay variation was below 12% between 32.6 and 1380 nmol/l. The inter-assay coefficients of variation between 18 assays were 14.6% at 37.2 nmol/l, 11.9% at 194.6 nmol/l and 11.8% at 680.3 nmol/l. The average detection limit of the assays was 6.3 nmol/l.

### Prostaglandin F_2α _metabolite analyses

The main initial blood plasma metabolite of prostaglandin F_2α_, 15-keto-13,14-dihydro-PGF_2α _(15-ketodihydro-PGF_2α_), was analyzed by radioimmunoassay [12]. The relative cross-reactions of the antibody were 16% with 15-keto-PGF_2α_, and 4% with 13,14-dihydro-PGF_2α_. The intra-assay coefficients of variation ranged between 13.1 and 13.0% for different ranges of the standard curve and the inter-assay coefficient of variation was around 11.8%. The practical limit of sensitivity for the assay analyzing 0.2 ml of plasma was 100 pmol/l.

### Statistical analysis

The cortisol and PG-metabolite values were analyzed with the repeated measurement analysis of variance using the MIXED procedure according to the Statistical Analysis System program package, version 9.1 (SAS Institute INC., Cary, NC, USA). The statistical model included treatment (n = 3), period (n = 6), the interaction between treatment and period, and the random effect of sows within treatment. The first period includes time -10, -5 and 0 minutes. The second period includes minutes 1 to 4 after service when stimulation either by the boar or human started. The third period includes 6 to 8 min, the fourth 10 to 20 min, the fifth 25 to 30 min and the sixth 35 to 50 minutes after service. Data are expressed as least squares means (LSmeans) ± SEM and probabilities less than 0.05 were considered significant.

## Results

Twenty-five out of 33 sows, which were used in this study, became pregnant and farrowed at full term. The evaluation of the results is from these 25 animals, 8 sows in Boar-group, 9 sows in IUI-Group and 8 sows in AI-Group. There were no differences in the number of total born piglets per litter (mean ± SEM) from sows mated, intrauterinally inseminated and conventionally inseminated (13.9 ± 0.5, 15.6 ± 1.3 and 13.1 ± 1.0, respectively).

### Cortisol

**Figure 1:**
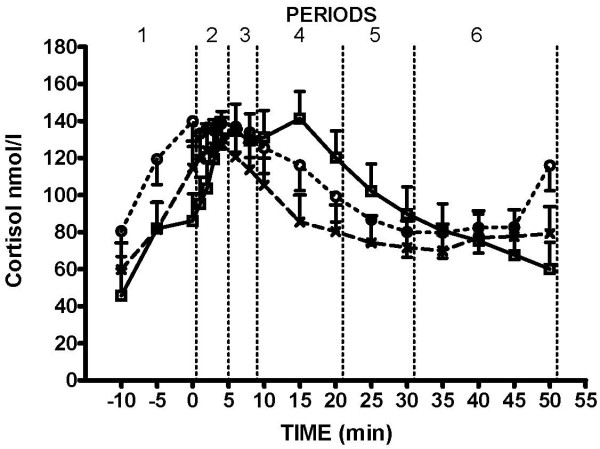
**Changes in plasma concentrations of cortisol**. Changes in plasma concentrations of cortisol (LSmean ± S.E.M.) in Boar-group (□), AI-group (x) and IUI-group (○). Time 0 (min) is the time when service commenced either by the boar or human stimulation.

Before treatment (mating or insemination), concentrations of cortisol were higher (P < 0.008) in the IUI-group when compared to sows from the Boar-group (Fig. 1). In sows from the IUI-group, there was an increase in concentrations of cortisol from approximately 78 nmol/l at -10 min to 130 nmol/l at 1–2 min after service started. Afterwards, cortisol concentrations reached a plateau that lasted for 7–8 min. From 10 min after service started the concentrations of cortisol decreased gradually in AI- and IUI-group to approximately 75 nmol/l (Fig. 1). In the mated sows, maximum cortisol concentrations were approximately 144 nmol/l 15 min after service. During period 4 i.e. 10 to 20 min after service, cortisol concentrations were higher in the Boar-group than in sows from the AI-group (130.9 ± 11.3 vs. 90.4 ± 11.3 nmol/l, P < 0.05)

### Prostaglandin F_2α _metabolite

**Figure 2:**
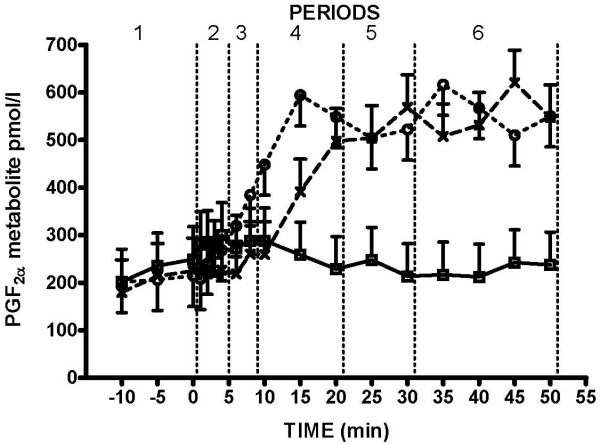
**Changes in plasma concentrations of PGF_2α _metabolite**. Changes in plasma concentrations of PGF_2α _metabolite (LSmean ± S.E.M.) in Boar-group (□), AI-group (x) and IUI-group (○). Time 0 (min) is the time when service commenced either by the boar or human stimulation.

In the Boar-group, there were no differences in PG-metabolite levels between periods (Fig. 2). In both IUI- and AI-group there was a significant increase of PG-metabolite concentrations in period 4 (P < 0.001), from about 220 pmol/l at the time when stimulation started (period 2). The IUI- and AI-groups had similar pattern in PG-metabolite rise but IUI- group started to rise earlier, already in period three (P < 0.01). As a result of this earlier increase, PG-metabolite concentrations in IUI-group were significantly higher than in AI-group during period four (530.5 ± 50.1 vs. 383.0 ± 53.2 pmol/l, P < 0.05). Afterwards, concentrations of PG-metabolite varied around 560 pmol in both IUI and AI-group (Fig. 2 – periods 5 and 6).

## Discussion

This study demonstrates that cortisol concentrations are higher in mated sows than in sows conventionally inseminated. On the other hand, both types of artificial insemination induce a dramatic increase of PG-metabolite, which is not seen in mated sows. It is known that mating of gilts results in a significant rise in plasma cortisol levels with a duration of approximately 30 min [13]. A significant transient increase in plasma concentrations of cortisol was also seen in oestrous gilts after introduction to the boar but not in gilts during the back pressure test [14]. In our previous study, we have shown that in sows housed in individual pens, concentrations of cortisol did not change after service and were significantly higher than in sows housed in crates before service [8]. Even though all sows were kept in crates in the present study, we have seen that sows in IUI-group had higher concentrations of cortisol before service than mated sows. The reason for high concentrations of cortisol might be due to fear of humans [15]. Thus, it is plausible that sows, which were randomly chosen for intrauterine insemination were more susceptible for handling before service. This could result in an early activation of the hypothalamic-pituitary-adrenal axis. Macaulay et al. [16] reported that in heifers an increase in plasma cortisol after mating was higher than after AI, with peaks after 15 and 60 minutes respectively. On the other hand in ewes there was no increase in cortisol during mating [17]. Houdeau et al. [18] made a comparable study on ewes, where plasma cortisol levels were measured during difficult and easy AI procedures. Their results showed the same outline independent of procedures, a quick but diminutive rise in plasma cortisol, reaching its maximum after 10 minutes, and with 30 minutes duration.

In inseminated sows from our previous study [8], basal level of PG-metabolite around 400 pmol/l and maximum values around 1000 pmol/l were seen. In the present study we found that the basal level of PG-metabolite was around 200 pmol/l and maximum values were around 560 pmol/l. We have no reasonable explanation for this discrepancy. However, it is important to know that the relative increase of PG-metabolite after service is similar i.e. approximately 2.5 fold. Claus et al. [19] reported that mating immediately increased PG-metabolite concentrations to a maximum 10 min after the onset of ejaculation, and this maximum coincided with maximal oestradiol concentrations in peripheral blood plasma. These authors suggested that oestrogens in seminal plasma, after natural service, lead to release of PGF_2α _from the endometrium and this might explain the increase of myometrial contractions. The finding of PG-metabolite elevations in sows after mating is in contrast to our present, as well as previous results [8,9]. At present, we have no reasonable explanation for this discrepancy. However, a dramatic increase of PG-metabolite in inseminated sows seen in our present, as well as previous studies [8,9] is in agreement with studies performed by Willenburg et al [4]. The inclusion of oestradiol in the extended semen dose prior to insemination did not result in a higher production of PG-metabolite (1 h after AI) as did vaginal deposition of oestradiol in corn oil compared to control inseminated sows. Taken together, there is a causal link between oestradiol inducing a uterine release of PGF_2α_, and an enhanced uterine contractility [4]. Wongtawan et al. [20] suggested that cervical stimulation during intrauterine insemination trigger the release of other hormones than oxytocin such as prostaglandins. In earlier studies, Langendijk et al. [21] measured myometrial activity in oestrous sows during infusion of different substances. These authors could not see any differences in contractions between saline and seminal plasma, but oestrogens, cloprestenol (prostaglandin analogue) and clenbuterol altered the contractions significantly. Cloprostenol increased whereas clenbuterol decreased both frequency and amplitude of uterine contractions. Both suppression and stimulation of uterine contractility might adversely affect fertilization in sows [5]. These authors concluded that new studies are required to establish an optimal level of uterine contractility and to investigate the importance of the timing of uterine contractility relative to the moment of insemination. This is in line with studies by Cao et al. [2], who reported that low concentrations of prostaglandins stimulate and high concentrations inhibit contractions of porcine myometrium. It is also plausible to speculate that these effects depend on the presence of contractile and relaxant prostanoid receptors in the cornua of the porcine uterus [22].

Semen transport to the site of fertilization in pigs is not only dependent on myometrial contractility but also on the spontaneous motility of the oviduct [23]. Intraluminal pressure in the porcine oviduct was not affected by saline, oestrogen, boar seminal plasma or boar semen. However, after deposition of oestrogen solution in the uterus and AI with 100 ml of boar semen, a clear increase of PG-metabolite could be seen [24,25]. An exogenous administration of PGF_2α _increased the peripheral plasma levels of PGF_2α _metabolite and produced an increase in the frequency of the phasic pressure fluctuations in the porcine oviductal isthmus [26]. Interestingly, Rodriguez-Martinez et al. [27] reported that concentrations of PGF_2α _in the oviductal fluid were high during the first day and reached a maximum during the 2^nd ^day of oestrus in gilts. These authors concluded that the collection catheter and/or manipulation at surgery have a clear effect on the prostaglandin F_2α _production and release. Therefore, a dramatic increase of PG-metabolite seen in intrauterine and conventionally inseminated sows might be associated with the insertion of the catheter. The other plausible explanation for such dramatic increase of PG-metabolite might be the effect of semen extender, for example, as EDTA used in our study. Findings of Willenburg et al. [4] might indicate that the extender that was used in their study also stimulate production of PG-metabolite in inseminated sows.

An intravenous injection of CRH results in elevated cortisol levels, with a peak reached at 20 min without any effect on plasma PG-metabolite levels in cycling gilts or in castrated boars [28]. In contrast to CRH-treated pigs, an increase of PG-metabolite followed by a cortisol peak approximately 40 min later were seen after the administration of ACTH in ovariectomized gilts, cycling gilts and castrated boars [9,28]. In the present study cortisol levels were elevated earlier than PG-metabolite in AI-groups, which might suggest that ACTH is not involved in elevating cortisol levels during AI. Furthermore, a study by Montgomery et al. [29] showed that injection of ACTH gives a peak in cortisol after 4 hours and it takes 9–10 hours before cortisol levels are back to a basal stage. Lang et al. [30] reported that it takes one hour for the cortisol to reach a maximum and one hour more for cortisol to return to basal levels after ACTH injections in sows. However, our results demonstrate a rise in plasma cortisol level almost immediately, with a peak 15 minutes after service. Observations have been made that CRH, can possibly act directly or indirectly to increase cortisol secretion further than that accomplished by ACTH on the adrenal gland [31]. Furthermore, the splanchnic nerve is capable of increasing the cortisol levels. In a well-designed study on calves, Edwards and Jones [32] found roughly the same pattern and duration of the cortisol release when they stimulated the splanchnic nerve as we found during AI and mating, probably due to a CRH-like peptide released from the adrenal medulla. These authors have also shown that the production of cortisol during intravenous infusion of ACTH could be further increased after stimulation of the splanchnic nerve [33]. Van Oers et al. [34] demonstrated that administrated CRH most likely increase the adrenal cortex sensitivity to ACTH due to a higher blood flow through the adrenal gland, and these authors hypothesized that intra adrenal CRH is controlled by the central nervous system.

It might be of importance to look upon our results with some knowledge about corticosteroid-binding globulin (CBG) and the relation between the total and free cortisol. Both total and free corticosteroid plasma levels were increased after mating in gilts, with significant changes in the maximum corticosteroid binding capacity [5]. Nyberg and Madej [35] reported that an anovulatory oestrus in sows seemed to be associated with high CBG binding capacity and high cortisol level in blood plasma, which might result in pronounced reduction of circulating free cortisol. Furthermore, studies of Klemcke et al. [36] demonstrated the occurrence and changes of CBG-like activity and cortisol content in porcine uterine lumen during the oestrous cycle. Endometrial CBG-like activity and free cortisol could indicate their biological role on uterine function in pigs.

At the present time, we have no obvious explanation for the biological effect of cortisol during the very short time for mating or insemination. Because of the well-known anti-inflammatory effects of glucocorticoids [37], it is plausible that the significant elevation of cortisol concentrations in mated sows may have an impact on the inhibition of prostaglandin synthesis. Cortisol metabolism and differential regulation can occur within different peripheral target tissues [38] and glucocorticoids are known to be involved in female reproductive function [39]. In a study on bovine endometrium by Lee et al. [39], it is concluded that cortisol inhibits both TNFα-stimulated PGE_2 _and PGF_2α _as well as basal PGF_2α _production in stromal cells. Interestingly, cortisol does not affect PGE_2 _production in stromal cells, nor OT-stimulated PG production in epithelial cells. Since the endometrium consists of many more stromal than epithelial cells, cortisol-inhibited PGF_2α _production could be of physiological relevance. With this in mind it could be of importance to look at 11β-hydroxysteroid dehydrogenase (11-HSD), an enzyme with two isoforms that facilitate a conversion between the active cortisol and inactive cortisone [38]. At least in heifers the isoform 11-HSD1, converts cortisone to cortisol, and has greater activity during the late follicular stage and oestrus [39]. Since our results indicate a hormonal difference between mating and insemination, either intrauterinally or conventionally, the reproductive outcome could be affected by the service technique, as it was shown by Tummaruk et al. [40] with data from almost 7000 sows that naturally mated sows had 0.3 more piglets born alive per litter than AI sows.

## Conclusion

We have shown that mating gives a greater increase of cortisol than AI without the amplified PG-metabolite plasma level seen in both the inseminated groups. It was also demonstrated that IUI-group had an earlier significant increase of PG-metabolite concentrations than sows inseminated conventionally. Further investigation using different semen extenders or even different type of insemination catheters might be helpful in understanding the reason for an immediate increase of PG-metabolite after insemination but not after mating.

## Competing interests

The author(s) declare that they have no competing interests.

## Authors' contributions

MN was responsible for hormones and data analyses, and manuscript preparation. MTM was responsible for the study design and collection of samples. CBA participated in the study design and collection of samples. HK contributed with expertise in prostaglandins. AM was involved in the study design and responsible for revision of the manuscript. All authors read and approved the final manuscript.
